# Unexpected hair regrowth in a woman with longstanding Alopecia universalis

**DOI:** 10.3205/oc000181

**Published:** 2021-05-11

**Authors:** Fani Akritidou, Konstantina Misiou, Elina Tsanidou, Georgios N. Katsaras, Theodora Papamitsou

**Affiliations:** 1 Ophthalmology Department, General Hospital of Pella – Hospital Unit of Edessa, Greece; 2 Paediatric Department, General Hospital of Pella – Hospital Unit of Edessa, Greece; 3 Department of Histology-Embryology, Aristotle University of Thessaloniki, Greece

## Abstract

Alopecia areata (AA) is an autoimmune disorder leading to non-scarring hair loss. As long as hair follicles are not destroyed, the potential for hair regrowth remains. Alopecia universalis is a severe form of AA and the chance of full hair regrowth is below 10%. We present a case of a 55-year-old woman with longstanding AA, who presented to the Outpatient Clinic of our Hospital Unit with almost full eyelashes and hair regrowth after an emotional stressful event. She reported no hair regrowth for the last 35 years. There are few case reports which have exhibited that hair follicles are still alive and may be reactivated after many years.

## Case description

A 55-year-old woman presented at our department with a complaint of foreign body sensation, redness and itching in both her eyes for the past 3 weeks.

The patient was diagnosed with Alopecia areata (AA) at 18 years of age and progressed to Alopecia universalis within a year. She reported complete eyelash loss, with upper and lower eyelid involvement in both eyes. Since the age of 20 years old, despite topical and systemic treatments, there was no hair regrowth. The patient was not able to recall specific details of the treatments received. Her condition was well accepted by her husband and family and she had a normal social life and a healthy self-esteem.

During these years, extended diagnostic work-up was performed; thyroid and autoimmune diseases, anemia and other dermatological diseases were ruled out. The patient’s past medical history revealed recurrent first-trimester miscarriages, with no specific causes found in the work-up. In the last 2 years, she has been in asymptomatic menopause transition.

**Figure 1:**
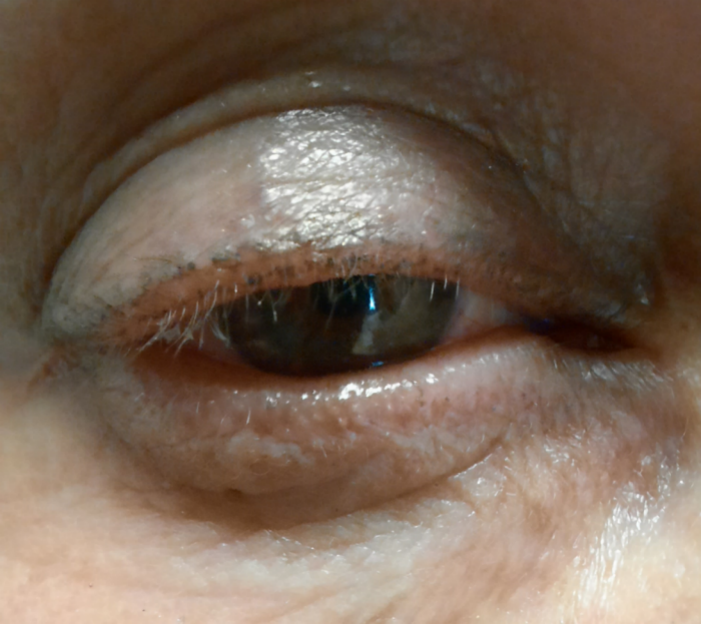
Eyelashes regrowth, right eye

**Figure 2:**
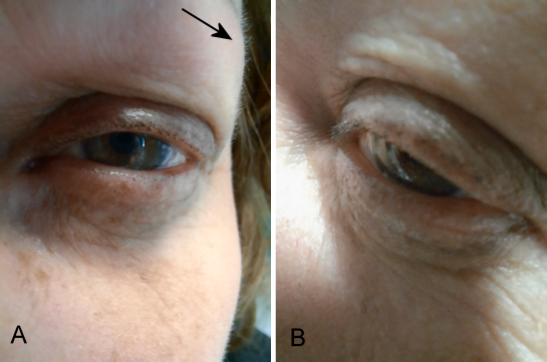
Left eye, A) facial hair regrowth (black arrow) and B) eyelashes regrowth

The patient’s ocular history was remarkable. Slit-lamp examination revealed eye-lashes regrowth. At a second follow-up visit after 2 months, almost full regrowth of eyelashes was observed in her right eye (Figure 1 (Fig. 1)) and partial regrowth was described in her left eye (Figure 2 (Fig. 2)).

The patient reported a stressful event (sudden loss of her husband) six months ago and a complete change in her daily activities, as she had to take over a family business and deal with many practical issues.

She was referred to a dermatologist for further evaluation, and hair regrowth was confirmed on the scalp and body.

Although the potential for regrowth of hair in patients with Alopecia areata, even in the severe form of Alopecia universalis, is retained [1], this is an interesting case because hair regrowth is occurring after 35 years. In addition, hair regrowth was probably triggered by a stressful event. Although our patient is a well-balanced person, capable to deal with practical and everyday issues, grief was the only trigger that could be related to this sudden hair regrowth. Even though psycho-emotional stress and hair loss can be related [2], the exact mechanism of interaction is still not fully understood.

## Discussion

Alopecia areata (AA) is an autoimmune disorder causing non-scarring hair loss. Although the exact pathophysiology remains unclear, abnormal antibodies directed to hair follicle antigens have been found [3].

There are three main types of Alopecia areata (AA) [4]:

- Alopecia areata (patchy),
- Alopecia totalis: hair loss across the entire scalp,
- Alopecia universalis: hair loss across the entire scalp and face (including eyebrows and eyelashes) and the rest of the body.

The precise event that triggers alopecia areata remains unknown. Most commonly, the disease is precipitated by emotional or physical stress, vaccines, viral infections, and drugs [5].

In AA the inflammation does not destroy hair follicles, thus the potential for hair regrowth remains. In Alopecia totalis (AT) and Alopecia universalis (AU), which are severe forms of AA, the chance of full hair regrowth is infrequent and is less than 10% [6], [7]. Important prognostic factors are the extent of hair loss and patient age at disease presentation [1].

Available therapeutic options include intralesional and topical corticosteroids as first-line treatment in patchy AA, while more severe cases can be treated with topical immunotherapy. As second line therapies minoxidil, anthralin, and psoralen + ultraviolet light A (PUVA) can be used. Systemic therapies include systemic corticosteroids, methotrexate, cyclosporine, azathioprine, and etanercept, but are reserved only for patients with AT [8]. It is difficult to evaluate the efficacy of current treatments due to the possibility of spontaneous remission and the fact that clinical responses may vary a lot among patients [9].

## Notes

### Informed consent

Informed consent has been obtained from the patient for the publication of this case report.

### Competing interests

The authors declare that they have no competing interests.
